# Effect of antiretroviral therapy on all-cause mortality among people living with HIV/AIDS in Ghana using Mahalanobis distant metric matching within propensity score caliper analysis: A retrospective cohort study

**DOI:** 10.1371/journal.pone.0203461

**Published:** 2018-09-07

**Authors:** Duah Dwomoh, Chrisantus Tambaa, Stephen Ayisi Addo, Ekow Wiah, Marijanatu Abdulai, Samuel Bosomprah

**Affiliations:** 1 Department of Biostatistics, School of Public Health, University of Ghana, Accra, Ghana; 2 Ghana Health Service, Accra, Ghana; 3 National AIDS/STI Control Programme, Accra, Ghana; University of KwaZulu-Natal, SOUTH AFRICA

## Abstract

Several health interventions have been put in place to improve health outcomes of people living with HIV/AIDS (PLHIV) in Ghana. We evaluated the impact of Antiretroviral Therapy (ART) on all-cause mortality in Ghana using matching procedures. This was a retrospective cohort study of 12,881 HIV/AIDS patients initiated on ART at 40 sentinel sites and 199 treatment centers between 2013 and 2016 countrywide. Patients were included if they had date of ART initiation and if they had mortality outcome recorded. Mahalanobis distant metric matching within propensity score caliper and other matching procedures were used to evaluate the effectiveness of ART in reducing the risk of all-cause mortality among PLHIV in Ghana. We performed sensitivity analysis using different matching procedures including Kernel weighting adjustment and Mahalanobis distance metric matching with nearest neighbour to ascertain the robustness of our results in the presence of unmeasured covariates. The proportion of patients on ART was 60.3% (95% CI: 59.5–61.1). The total number of mortalities reported was only 734 representing 4.6% (95% CI: 4.2–4.9) of the studied population. The risk of all-cause mortality has reduced by 11.6 percentage point among HIV/AIDS patients who were on ART compared to those who were not on ART (95% CI: 9.6–13.4). ART was associated with a decreased risk of all-cause mortality. Effort being made by Government and non-Governmental organizations in support of ART treatment in Ghana should continue unabated to help reduce mortality rate and improve health outcomes among HIV/AIDS. To reduce bias to the barest minimum between treatment and intervention group when evaluating the effectiveness of health interventions, it is recommended to use matching procedures especially when the study design is not a randomized control trial.

## Introduction

Human Immunodeficiency Virus (HIV) is a life threatening chronic viral infection, which compromises the body’s immune system [1]. The virus renders infected persons susceptible to other infections usually referred to as Opportunistic Infections (OI). People Living with HIV (PLHIV) are therefore susceptible to other diseases that uninfected persons may usually not have. It is estimated that over 36 million people, worldwide, are living with HIV of which most are in Sub-Saharan Africa as at the year 2015 [1].

HIV has, since its diagnosis in Ghana in 1986, recorded 3.8% (250,232) of the 6.5 million cases in Western and Central Africa with adult prevalence rate of 1.47% as at December 2015 [2], [3]. The National AIDS/STI Control Programme (NACP), Ghana Health Service 2015 HIV Sentinel Survey report indicated that HIV type 1 is the most prevalent in the country. The infection is reported to be more prevalent in urban areas, border towns, among female commercial sex workers, men having sex with men and among people within 45–49 years age group [2], [3]. HIV & AIDS related mortality is persistently among the leading causes of mortalities in Ghana [3]^,^[4]^,^[5]. Causes of mortalities could be attributed to late diagnosis and initiation of treatment, poor access to healthcare, TB/HIV co-infection and poor environmental factors which increase the risk of opportunistic infections in resource poor countries[6]^,^[7].

Antiretroviral Therapy (ART) was introduced in Ghana in 2003 and assessing its impact on mortality is an important consideration. Several studies have assessed the impact of ART on mortality but these studies have relied primarily on traditional regression models (logistic, Poisson and Cox proportional hazard models). These traditional models have been shown to lead to biased results when the covariate distributions in the groups are very different[8–10]. Selection and regression models have been shown to perform poorly in situations where there is insufficient overlap among covariates, but their standard diagnostics do not involve checking this overlap[11–13]. However, matching procedures have straightforward diagnostics by which their performance can be assessed (Stuart, 2010). Matching procedures mirror randomized experiment as closely as possible using techniques that make PLHIV who are on ART and those who are not on ART as similar as possible on covariate distributions. This then reduces bias due to the observed covariates.

Motivated by nationally representative HIV and AIDS data, we illustrated how to use matching procedures and sensitivity analysis in evaluating causal effect of ART. Specifically, we evaluated the effectiveness of ART on all-cause mortality for 2013–2016 cohort using propensity score matching and Mahalanobis distance metric matching with propensity score caliper. The latter have been proven to yield matches that are relatively well matched on the propensity score and particularly well matched on Mahalanobis covariates especially when the continuous covariates used in generating the Mahalanobis distance are well matched [14].

## Methods

### Ethical statement

The study was approved by the Institutional Review Board of Noguchi Memorial Institute for Medical Research (NMIMR) of the University of Ghana, Accra. The National AIDS/STI Control Programme (NACP) gave the authorisation to use the data. The personal identification code which include name of the HIV patient and the health facility where the subject obtained ARV therapy were excluded from the data before data were handed over to the authors. That is, the data were de-identified and therefore cannot be linked to any patients.

### Study design and data management

This was a retrospective cohort study of HIV patients enrolled in 40 sentinel sites and 199 treatment centres from 2013 to 2016 in the NACP national database. NACP is a Ghana Health Service agency under the ministry of health that captures, processes and stores the country’s data on HIV. NACP collates data on services provided to HIV patients through 40 sentinel sites and 199 treatment centers countrywide. In Ghana, patients are scheduled monthly for services at allocated treatment centers across the country. Each treatment center is equipped with data capturing tools including patient folders, computers and an offline database where registration records and updates on treatment from visits are entered daily by trained data managers or by nurses and supervised by a zonal data manager. Data is stored on a server at the national level and updated quarterly with databases from the treatment centers. NACP uses a Microsoft access database and application program to collect and process data at all levels (treatment centers, regional and national).

The entered data is validated by a regional and national team and copy taken by the team to the national database on quarterly basis. The national data repository comprises data on all HIV patients captured and reported by all treatment centers. The data collected at community or treatment centers are validated and sent by electronic mail in a pass worded database form to the data manager at the national level where they are merged and stored in the server. The data for this study was extracted from the national database into excel for preliminary cleaning, error entry and assessment of missing values. A total of 16,311 HIV/AIDS patients data were extracted from the database within the study period. Patients were included if they had date of ART initiation and if they had mortality outcome recorded as died or alive. A total of 12,881 patients were eligible for analysis in this study. The excel data was then imported into Stata 14 (StataCorp, College Station, Texas, USA) for further cleaning and statistical analysis. Table 1 shows the variables used in the study.

**Table 1:** Description of the outcome variable and covariates used in the study.

Covariates	Type of variable	Description	Scale of measurement	Measurement
Mortality	Outcome	Whether a patient dies or censored due to lost to follow-up or is alive at the end of the study period	Categorical	Dead or censored
Age at entry	Outcome	The age of the patient at registration	Continues categorical	Number of years as continuous measure and categorized as (<15, 15–35, 36–59, 60+)
Initial cd4 cells count	Explanatory	The count of CD4 cells of the patient at first examination	Discrete categorical	Count of CD4 cells per cubic millimetre (< = 200, 200–349, 350–499, & > = 500)
HIV clinical stage at entry	Explanatory	The stage of the infection at diagnosis	Categorical	Asymptomatic (stage I) Mild symptoms (stage II) advanced symptoms (stage II) & severe symptoms (stage IV)
HIV/TB co-infection	Explanatory	The patient has both HIV and TB infections	Binary	Yes, No
HIV type	Explanatory	The type of HIV infection the patient is suffering from	Binary	Type I, Type II
Sex	Explanatory	Whether a patient is a male or female	Binary	Male, Female
Educational status	Explanatory	The highest education a patient has had as at registration	Categorical	None/primary/JHS.MSLC/SHS.STS/tertiary
Marital status	Explanatory	Whether the patient has been married or not	Categorical	Single/married/widow(er)/ Divorced/cohabiting

### Statistical analysis

The primary outcome of interest was all-cause mortality defined as all of the death that occurred in the sample regardless of the cause. We compared baseline sociodemographic characteristics and biomarkers between patients who are on ART and patients who are not on ART using Pearson’s chi-square test of independence and Cochran Armitage trend test where appropriate. Modified Poisson and Cox-proportional hazard models with robust standard errors were used to estimate effect of ART (defined as the percentage reduction in the number of mortalities as a result of ART calculated as 100 (1 − *R*), where *R* is the hazard ratio estimated by Cox regression or incidence rate ratio estimated by Poisson regression models (Cheung et al. 2010). Date of treatment initiation was considered as when the patient became at risk of death and was therefore set as the origin in calculating the survival time. Patients were considered as censored at the date they were last seen without the outcome (i.e. lost to follow up) or at the end of data cut-off point without having the event of death. We elected one year data cut-off point for this study. The modified Poisson and Cox-proportional hazard models were used for purposes of comparison of estimates from traditional methods with those from the matching procedures.

We estimated average treatment effect among PLHIV (ATET) using Mahalanobis matching within propensity score callipers proposed by Rubin and Thomas (2000). The propensity score for *ith* patients was then defined as the probability of receiving the ART treatment given the observed covariates based on the probit regression model. Thus, the probability to receive ART given the observed covariates was given by *e*_*i*_(*X*_*i*_) = *P*(*ART*_*i*_|*X*_*i*_) where *e*_*i*_(*X*_*i*_) is the estimated propensity score, *X*_*i*_ represent individual’s age in years, baseline CD4 count (cells/mm3), educational level, sex, marital status, clinical stage of the disease at diagnosis and time on treatment.

The Mahalanobis matching within propensity score callipers proposed by Rubin and Thomas [15] defines the distance between patients *i* and *j* as:

$$D_{ij}=\left\{ \begin{array}{l} {\left(Z_{i}-Z_{j}\right)}^{\prime }\Sigma^{\prime }(Z_{i}-Z_{j})\;if\;|logit(e_{i})-logit(e_{j})|\leq c\\ \;\infty \;if\;|logit(e_{i})-logit(e_{j})|>c \end{array}\right.$$

where *c* is the caliper, *Z* is the age and baseline CD4 count before treatment, Σ is the variance covariance matrix of *Z*.

We used numerical and graphical diagnosis to evaluate the common support of the distribution of propensity score between patients on ART and those who are not ART. We compared the multidimensional histograms and Kernel density plots of the covariates in the matched ART and non-ART groups. We assumed that the standardized differences greater than 10% in absolute value indicate serious imbalance in the covariate of interest between the two groups[16]. The standardized difference *d* is given as follows for continuous and binary indicator variables:

$$d_{cont}=\frac{\left({\bar{X}}_{ART\;patient}-{\bar{X}}_{non-ART\;patient}\right)}{\sqrt{\frac{S_{ART\;}^{2}+S_{Non-ART}^{2}}{2}}}\times 100\%;d_{binary}=\frac{\left(P_{ART\;ppatient}-P_{non-ART\;patient}\right)}{\sqrt{\frac{P_{T}(1-P_{T})+P_{c}(1-P_{c})}{2}}}\times 100\%$$

where *S* is the standard deviation of the continuous covariate, *P*_*T*_ is the proportion of patients on ART, *P*_*C*_ is the proportion of patients who are not on ART.

### Sensitivity analysis of matching procedures

We also performed sensitivity analysis of the ignorability assumption under propensity score matching. This assumption states that there may be other unobserved factors that influence ART treatment enrollment, all-cause mortality or both. We calculated the Rosenbaum bounds for average ART effects on the treated in the presence of unobserved heterogeneity (hidden bias) between patients who are on ART and those who are not on ART[9]. We also assessed whether the effectiveness of ART were robust to the type of matching procedure used. Since different matching technique could produce slightly different results (impact estimates), Kernel weighting adjustment and Mahalanobis distance metric matching with nearest neighbour were performed to confirm the robustness of the ART estimate. We further compared our impact estimate of ART with results that could have been obtained from various regression adjustments including Cox-proportional hazard model and the modified Poisson regression with robust standard error. The proportional hazard assumption required for the Cox-proportional hazard model was tested using the Schoenfeld residuals. We further tested for over-dispersion of our outcome variable under the Poisson regression model. All analyses were performed using Stata 15 (StataCorp, College Station, Texas, USA).

## Results

### Description of the study participants

A total of 12,881 patients were included in the analysis. Of these, 74.2% were females and 25.8% were males. The median age was 37 years (IQR = 30, 45) (Table 2) with the youngest person aged 1 year and oldest aged 92 years. More than half (56.7%) of the patients were married with only 925 (5.8%) having attained tertiary education. Almost all the patients were HIV type 1 (96.9%) with 71% being TB positive. The median CD4 count at baseline was 245 (IQR *= 90*, *445*) cells per cubic millimeter. The standard WHO classification of stage of the infection at diagnosis is as follows: stage I (33.3%), stage II (17.7%), stage III (44.0%) and stage IV (5.1%) (Table 2). We have a total of 385,247.5 persons-month at risk and the longest follow up was 48.7 months. The median time at risk of mortality in month is 23.6 months (IQR = 10.7, 36.5). The median duration of treatment is 18.6 months. With the exception of HIV type (p = 0.648) and TB/HIV coinfection status (p = 0.641), patients on ART were significantly different from those who were not ART in terms of age (p<0.001), sex (p = 0.002), educational attainment (p<0.001), marital status (p<0.001), CD4 count at baseline (p<0.001) and clinical stage of the disease (p<0.001) (Table 2).

**Table 2:** Comparing baseline characteristics of study participants by antiretroviral therapy status.

Predictors of ART	ART: N (%)	Non ART: N(%)	P-value estimate from the Chi-square test statistic
Sex (N = 12810)			0.002**
Male	1849(57.9)	1340(42.1)	
Female	3770(38.9)	5921(61.1)	
Age in years (N = 12801): Median (IQR)	37 (30, 45)	35 (28, 43)	
Age in years categorized			<0.001***
<15	58(70.7)	24(29.3)	
15–35	3435(56.5)	2647(43.5)	
36–59	3918(63.7)	2235(36.3)	
60+	299(61.8)	185(38.2)	
*Educational status (N = 12486)*			<0.001***ǂ
No formal education	1648(58.1)	1187(41.9)	
Basic	4465(60.1)	2964(39.9)	
Secondary	961(65.3)	511(34.7)	
Tertiary	482(64.3)	268(35.7)	
*Marital status (N = 12530)*			<0.001***
Unmarried	2592(61.9)	1595(38.1)	
Married	4194(58.3)	2997(41.7)	
Widow(er)	763(66.2)	389(33.8)	
**Biomarkers**			
*HIV type (N = 11416)*			0.648
HIV-1	6689(60.3)	4396(39.7)	
HIV-2	44(61.9)	27(38.1)	
HIV-1&2	164(63.1)	96(36.9)	
Clinical stage of HIV at diagnosis			<0.001***ǂ
Stage I	2433(60.8)	1567(39.2)	
Stage II	1380(63.6)	789(36.4)	
Stage III	3433(59.7)	2316(40.3)	
Stage IV	321(52.5)	290(47.5)	
*TB/HIV co-infected (N = 489)*			0.641
Positive	202(59.2)	139(40.8)	
Negative	91(61.5)	57(38.5)	
CD4 cell count: Median (IQR)	37(30, 45)	35(28, 43)	
CD4 cell count: categorized			<0.001***ǂ
<200	1266(57.2)	947(42.8)	
200–349	769(66.9)	381(33.1)	
350–499	489(68.7)	223(31.3)	
500+	5243(59.6)	3558(40.4)	

ART: Antiretroviral therapy, P-value notation

*** p<0.001

** p<0.01

*p<0.05

ǂ p-value estimate from the Cochran Armitage trend test; N(%): Frequency and row percentages.

### Effect of ART on all-cause mortality using modified Poisson and Cox regression models

The proportional hazard assumption test based on Schoenfeld residuals showed that there was no violation of this assumption (*χ*^2^ = 10.8,*p* = 0.544). Besides, the likelihood ratio test showed that there was no statistically significant difference between the conditional variance and the conditional mean (p = 0.899) which authenticate the used of Poisson regression model. A total of 548 (4.3%) out of the 12,881 patients died during the study period (Table 3). 13 (0.2%) out of 7,771 on ART died compared to 535 (10.5%) out of 5110 not on ART died (Table 3). In multivariable analyses, the hazard ratio estimated using modified Poisson regression model was 40.0 (95% CI: 17.7–90.2) compared to 52.3 (95% CI: 21.5–126.9) as estimated by Cox Proportional Hazard model (Table 3). All analyses adjusted for duration on ART, age, sex, CD4 count at diagnosis, stage of HIV at diagnosis, educational attainment and marital status.

**Table 3:** The effect of ART on all-cause mortality: Comparing modified Poisson and Cox proportional hazard models.

Exposure status	Number of patients (% of total) n = 12,881	Number of patients (% who died) d = 548(4.3)	Modified Poisson model				Cox Proportional Hazard Model			
			Crude IRR (95% CI)	P-value	^1^Adjusted IRR (95% CI)	P-value	Crude HR (95% CI)	P-value	^1^Adjusted HR (95% CI)	P-value
Not on ART	5110 (39.7)	535 (10.5)	ref		ref		ref		ref	
On ART	7771(60.3)	13 (0.2)	46.0 (26.5–79.7)	p<0.001	40.0 (17.7–90.2)	p<0.0001	56.2 (31.7–99.7)	p<0.001	52.2 (21.5–126.9)	p<0.001
Effect of ART (1-R): Percent reduction in mortality attributable to ART					390 (8920–1640)				5130 (12590–2050)	

ART: Antiretroviral therapy; IRR incidence rate ratio from the Poisson regression model with robust standard error, IRR: Incidence Rate Ratio, HR: Hazard ratio from the Cox-proportional hazard model; CI: Confidence interval. TB/HIV coinfection dropped because very few patients (489 out of 12881) had information on coinfection ^1^Estimates were adjusted for, duration on ART, sex, age in years, education, clinical stage of the disease at diagnosis and CD4 cell count per cubic millimetre, ref: Reference category, R: Hazard or the incident rate ratio from the Cox-proportional hazard and Modified Poisson regression models respectively.

### Matching procedures

**Fig 1:**
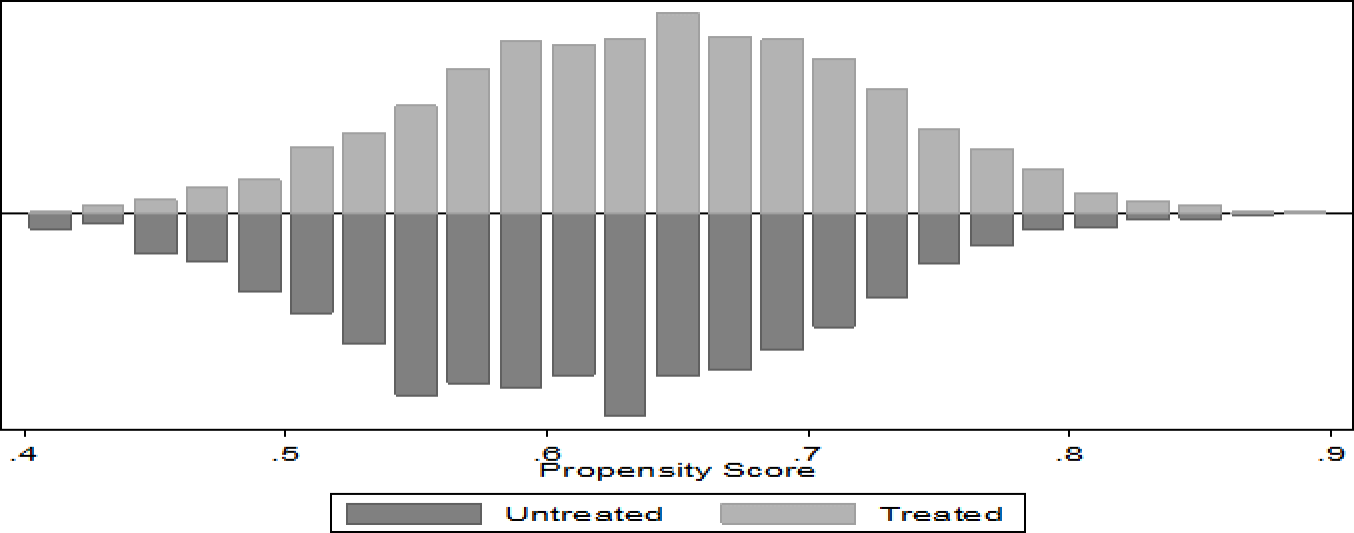
Assessing common support assumption required for propensity score matching procedure.

The average probability for patients to receive ART was 63.1% (95% CI: 62.9–63.3). Using the propensity-score with nearest-neighbour matching it was possible to generate a control group (non-ART) which was similar enough to the treatment group (patients on ART) to be used in estimating the average ART treatment effect on patients who actually received treatment. Fig 1 shows that there is an overlap of the propensity scores of the patients who are on ART and those who are not on ART which clearly shows that the assumption of common support holds in this study.

**Fig 2:**
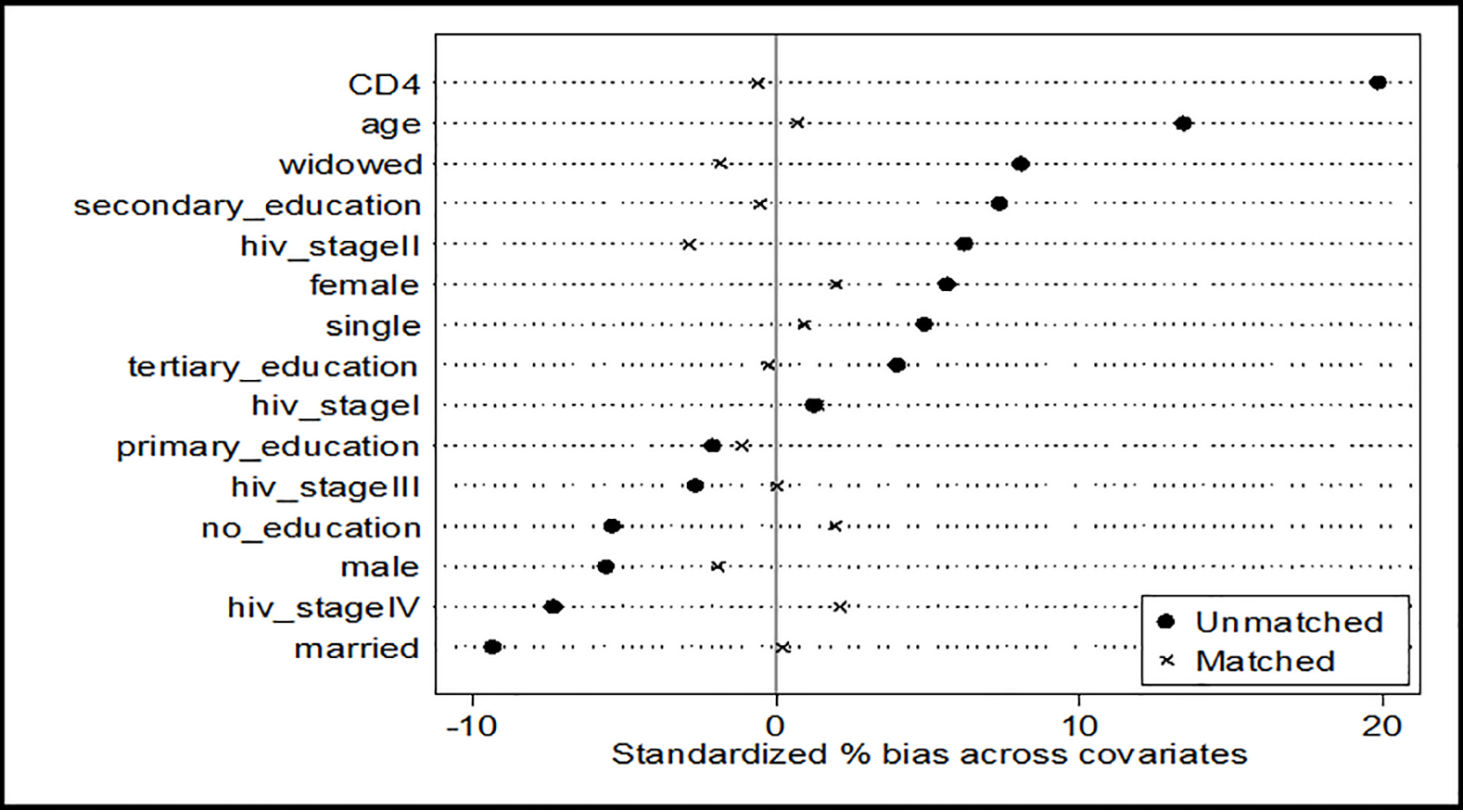
Covariate balance between patient on ART and non-ART patient using standardize percentage bias across covariates.

The diagnostic graph assessment of the covariate balance between ART and non-ART patients showed that standardized percentage bias among covariates between the two groups reduced drastically after matching (Fig 2).

Covariates distributions were found to be similar in both groups after matching. For instance, bias in CD4 count before matching between patients on ART and those not on ART was 19.8% but reduced to 5.8% after matching. Similar bias reduction were observed for other covariates. The overall mean bias before matching was 6.90% and this reduced 1.9% after matching as shown in Table 4.

**Table 4:** Standardized differences between patients on ART and non-ART patients.

	Before matching				After matching				
Variable	ART patients	Non-ART patients	Bias (%)	p-value	ART patients	Non-ART patients	Bias (%)	p-value	*% rABS(Bias)*
*Male*	0.24	0.26	-5.60	0.002**	0.27	0.28	-1.9	0.496	66.60
*Female*	0.76	0.74	5.60	0.002**	0.73	0.72	1.9	0.496	66.60
Age in years	38.08	36.60	13.40	<0.001	38.97	38.89	0.80	0.777	94.30
*No education*	0.22	0.24	-5.40	0.003**	0.18	0.18	-0.0	0.254	99.60
*Basic*	0.59	0.60	-2.10	0.254	0.59	0.60	-1.20	0.654	42.60
*Secondary*	0.13	0.10	7.40	<0.001	0.15	0.14	3.40	0.249	53.40
*Tertiary*	0.06	0.05	4.00	0.030*	0.07	0.08	-2.10	0.484	47.10
*Single*	0.34	0.32	4.90	0.007**	0.37	0.36	1.30	0.623	72.70
*Married*	0.56	0.60	-9.30	<0.001	0.53	0.53	-2.10	0.430	77.20
*Widowed*	0.10	0.08	8.00	<0.001	0.11	0.10	1.50	0.609	81.80
*Stage-I*	0.32	0.32	1.20	0.495	0.31	0.30	1.16	0.245	-146.00
*Stage-II*	0.18	0.16	6.20	0.001**	0.20	0.20	-0.40	0.885	-0.14
*Stage-III*	0.45	0.47	-2.60	0.149	0.44	0.45	-2.80	0.299	-5.00
*Stage-IV*	0.04	0.06	-7.30	<0.001	0.05	0.05	0.50	0.858	93.60
CD4	306.83	254.59	19.80	<0.001	307.75	292.50	5.80	0.038	70.80
Mean Bias		6.90				1.90			
Rubin’s B		36.5				8.00			
Rubin’s R		1.05				1.19			
LR, p-value		139.02,p<0.0001				9.10,p = 0.612			

Abbreviations: Rubin’s B: The absolute standardized difference of the means of the linear index of the propensity score in the treated (ART patients) and (matched) non-treated group (non-ART); Rubin's R: The ratio of treated (ART patients) to (matched) non-treated variances of the propensity score index. Rubin (2001) recommends that B be less than 25 and that R be between 0.5 and 2 for the samples to be considered sufficiently balanced. % rABS (Bias): Percentage reduction in absolute bias; p-value notation

*** p<0.001

** p<0.01

*p<0.05.

### Causal effect of ART on all-cause mortality

The different matching procedures used in this study showed that patients on ART have reduced risk of mortality compared to those who are not on ART (Table 5). PSM with 1:1 nearest neighbour matching showed that the risk of mortality if every patient is on ART is 10.9 (95% CI: 8.8–13.1; p<0.0001) less compared to if no patient is on ART (Table 5). Using Mahalanobis with 1:1 nearest neighbour, risk of mortality if every patient is on ART is 11.6 (95% CI: 9.6–13.4; p<0.0001) less compared to if no patient is on ART (Table 5). Similar results were obtained with 2:1 nearest neighbour and Kernel weighting adjustment (Table 5).

**Table 5:** Average ART treatment effect on all-cause mortality among HIV and AIDS patients in Ghana.

Matching procedures	ATET	95% CI	P-value
PSM with 1:1 nearest neighbour	-10.94	-13.10, -8.79	p<0.0001
PSM with 2:1 nearest neighbour	-10.30	-12.13, -8.48	p<0.0001
PSM with Kernel weighting adjustment	-11.00	-12.47, -9.44	p<0.0001
Mahalanobis with 1:1 nearest neighbour	-11.56	-13.38, -9.75	p<0.0001
Mahalanobis with 2:1 nearest neighbour	-11.59	-13.31, -9.86	p<0.0001
Mahalanobis within propensity score caliper	-10.93	-10.92,-10.01	p<0.0001

ATET: Average treatment effect on the treated (patients on ART); PSM: Propensity Score Matching.

### Sensitivity analysis to the ignorability assumption under PSM based on Rosenbaum (1983) bounds

Rosenbaum’s method of sensitivity analysis relies on the sensitivity parameter gamma (Γ) which measures the degree of departure from random assignment of ART. This assumption states that given the observed covariates, there are no unobserved differences between patients on ART and those not on ART (control group).

Two patients with the same observed characteristics may differ in the odds of receiving the ART by at most a factor of Γ. In a randomized experiment, randomization of the ART ensures that Γ = 1. In an observational study, if Γ = 1.5 and two patients are identical on matched covariates then one person might be 1.5 times as likely as the other to receive the ART because they differ in terms of an unobserved covariate[17]. Under the assumption of no hidden bias (Γ = 1), the Hodges-Lehmann test-statistic gives a similar result, indicating a significant ART effect on all-cause mortality (p<0.001). The median difference in mortality if there is no hidden bias is 11.0% lower for ART patients.

Looking at the bounds under the assumption that we have over-estimated the ART effect (upper bound Hodges-Lehmann point estimate) reveals that already at relatively small levels of Γ (1.1–2.5), the result becomes significant at alpha = 5% indicating no hidden bias. For instance for Γ = 2.5, mortality among patients receiving ART might be as low as (9.6%-12.4%) compared to those who are not on ART (Table 6).

**Table 6:** Sensitivity to the ignorability assumption under PSM based on Rosenbaum (1983) bounds.

Gamma (Γ)	Upper bound Hodges-Lehmann point estimate	Upper bound significance level	Upper bound confidence interval (α = .05)	Lower bound Hodges-Lehmann point estimate	Lower bound significance level	Lower bound confidence interval (*α* = .05)
**1.0**	-0.11	<0.0001	-0.11	-0.11	<0.0001	-0.11
**1.1**	-0.11	<0.0001	-0.11	-0.11	<0.0001	-0.11
**1.2**	-0.11	<0.0001	-0.11	-0.11	<0.0001	-0.11
**1.3**	-0.11	<0.0001	-0.12	-0.11	<0.0001	-0.10
**1.4**	-0.12	<0.0001	-0.12	-0.10	<0.0001	-0.10
**1.5**	-0.12	<0.0001	-0.12	-0.10	<0.0001	-0.10
**1.6**	-0.12	<0.0001	-0.12	-0.10	<0.0001	-0.10
**1.7**	-0.12	<0.0001	-0.12	-0.10	<0.0001	-0.10
**1.8**	-0.12	<0.0001	-0.12	-0.10	<0.0001	-0.10
**1.9**	-0.12	<0.0001	-0.12	-0.10	<0.0001	-0.10
**2.0**	-0.12	<0.0001	-0.12	-0.10	<0.0001	-0.10
**2.1**	-0.12	<0.0001	-0.12	-0.10	<0.0001	-0.10
**2.2**	-0.12	<0.0001	-0.12	-0.10	<0.0001	-0.10
**2.3**	-0.12	<0.0001	-0.12	-0.10	<0.0001	-0.10
**2.4**	-0.12	<0.0001	-0.12	-0.10	<0.0001	-0.09
**2.5**	-0.12	<0.0001	-0.12	-0.10	<0.0001	-0.09

Gamma (Γ): Log odds of differential assignment to ART due to unobserved factor; ART: Antiretroviral therapy, α = 0.05: Significance level.

## Discussion

This study was designed to evaluate the effectiveness of antiretroviral therapy on all-cause mortality among people living with HIV/AIDS in Ghana. The three years cohort study involved 12,881 HIV patients on ART (7,771) and not on ART (5,110), of which 4.3% (548) died within the period of study. Comparing these two groups, we found that those who were on Antiretroviral Therapy (ART) were of reduced risk of mortality. For example, our findings showed that the risk of mortality if every patient is on ART is 11.6 (95% CI: 9.6–13.4; p<0.0001) less compared to if no patient is on ART.

Our findings are consistent with what has been found across the globe. Lesko and colleagues [18] using national surveillance data in the USA, found that patients on ART had a reduced risk of mortality compared to those not on ART (5-year mortality risk difference = -17.7% (95%CI: -27.0%, -8.4%). Similarly, in a collaborative analyses of prospective cohort studies in South Africa, Europe and North America, Boulle and Colleagues [19]] also found that mortality was much higher in South Africa compared to Europe and North America but this reduced and reversed with longer duration on ART in South Africa [20–24].

We demonstrated the effectiveness of ART using various statistical procedures that compared the traditional methods of estimating effect of intervention and methods that minimises the effect of bias when evaluating the effectiveness of an intervention. The effect of ART on all-cause mortality estimated via the traditional procedures (Poisson and Cox-proportional hazard models) is 390% and 5130% respectively. A more robust measure of the effectiveness of ART was obtained after removing bias in the covariates. Patients on ART had 10.0–11.0 percentage points reduced risk of mortality compared to what was obtained using the traditional methods. This, suggests that the traditional regression models over-estimated the effectiveness of ART in reducing risk of mortality especially when the difference in the means of the propensity scores in the two groups being compared is large. As indicated by Cochran and Rubin [25], and Rosenbaum and Rubin [9], the three basic conditions that must generally be met for regression analyses to be trustworthy, in the case of approximately normally distributed covariates include: “the distributions of the covariates in both groups are nearly symmetric, the distributions of the covariates in both groups have nearly the same variances, and the sample sizes are approximately the same, the ratio of the variances of the propensity score in the two groups must be close to one (e.g., 1/2 or 2 are far too extreme), the ratio of the variances of the residuals of the covariates after adjusting for the propensity score must be close to one”. These conditions are rarely met in a non-randomized study design and therefore the use of matching procedures is more appropriate. It is important to note that matching methods and regression-based model adjustments should also not be seen as competing methods but rather as complementary. Austin, Cochran and Rubin [25], [26], Rosenbaum and Rubin [10] as well as Stuart [14] have all shown that the best approach is to combine the two methods. Regression analysis on those matched samples can adjust for small remaining differences and increase efficiency of our impact estimates as was the case in this study.

We highlight few limitations of this study. Several other factors might have contributed to all-cause mortality among the HIV/AIDs patients but the database used for this study did not have information on those variables and therefore the matching procedures excluded these unobserved variables. Matching methods assume that there are no unobserved differences between patients on ART and those who are not on ART (control group), conditional on the observed covariates. To satisfy this assumption, it is always advisable to include in the matching procedure all variables known to be related to both treatment assignment and the outcome[27],[28]. Although series of sensitivity analysis were conducted to assess the validity of this assumption, excluding important confounder could increase bias of impact estimate.

The study analysed secondary data that are pulled from all treatment centers into a national database and are thus subject to varying quality, incompleteness and under reporting. Also, not all events of HIV morbidity and mortality are captured at the treatment centres.

Nonetheless, we have illustrated the use of matching procedures in evaluating the causal effect of health intervention in an observational study.

Results from this study reiterate the importance of ART in reducing risk of all-cause mortality among HIV/AIDS patients. The various interventions put in place by Government and non-Governmental organizations in support of ART treatment in Ghana should continue unabated to help reduce mortality rate and improve health outcomes among HIV/AIDS patients in the country. There is the need to continue the education programme by NACP and its allied agencies on the importance of having continuous ART treatment for all HIV and AIDs patients in Ghana at all time.

## Conclusion

The use of causal inference methodologies such as Mahalanobis distant metric matching within propensity score caliper has shown efficient estimates of the effectiveness of ART in reducing all-cause mortality among people living with HIV. Future observational studies should consider use of these methodologies to enhance estimates from such studies.

## References

[1] UNAIDS, “Global AIDS Update,” pp. 1–16, 2016.

[2] P. A. Akwara, G. B. Fosu, P. Govindasamy, S. Alayón, and A. Hyslop, “An In-Depth Analysis of HIV Prevalence in Ghana,” 2005.

[3] Sturge C., “Commentary. [References].,” *Child Adolesc*. *Ment*. *Health*, vol. 11(1) 2, no. 2, pp. 46–48, 2006. 10.1111/j.1475-3588.2005.00385_2.x 32811053

[4] CDC, “HIV/AIDS Factsheet Impact in Ghana CDC in Ghana,” no. Cdc, pp. 1–2, 2010.

[5] WHO, “Ghana: Life Expectancy,” Ghana WHO Stat. profile, pp. 1–3, 2016.

[6] Manosuthi W., Chottanapand S., and Thongyen S., “Survival Rate and Risk Factors of Mortality Among HIV / Tuberculosis-Coinfected Patients With and Without Antiretroviral Therapy,” vol. 43, no. 1, pp. 42–46, 2006 10.1097/01.qai.0000230521.86964.86 16885778

[7] G. Y. Afful, “Factors influenicing low access to antiretrovirals for pregnant women living with HIV in Ghana to reduce mother to child transmission of HIV,” 2012.

[8] Baser O., “Choosing propensity score matching over regression adjustment for causal inference: when, why and how it makes sense,” *J*. *Med*. *Econ*., vol. 4, no. 10, pp. 379–391, 2007.

[9] Rosenbaum D. B., Rubin P. R., “The central role of the propensity score in observational studies for causal effects,” *Biometrika*, vol. 1, no. 70, pp. 41–55, 1983.

[10] Rosenbaum D. B., Rubin P. R., “Constructing a control group using multivariate matched sampling methods that incorporate the propensity score,” *Am*. *Stat*., vol. 1, no. 39, pp. 33–38, 1985.

[11] Dehejia S., Wahba R. H., “Causal effects in nonexperimental studies: Reevaluating the evaluation of training programs,” *J*. *Am*. *Stat*. *Assoc*., vol. 448, no. 94, pp. 1053–1062, 1999.

[12] Dehejia S., Wahba R. H, “Propensity score-matching methods for nonexperimental causal studies,” *Rev*. *Econ*. *Stat*., vol. 1, no. 84, pp. 151–161, 2002.

[13] Glazerman D., Levy S., Myers D. M., “Nonexperimental versus experimental estimates of earnings impacts,” *Ann*. *Am*. *Acad*. *Pol*. *Soc*. *Sci*., vol. 1, no. 589, pp. 63–93, 2003.

[14] Stuart E. A., “Matching methods for causal inference: A review and a look forward. Statistical science: a review,” *J*. *Inst*. *Math*. *Stat*., vol. 1, no. 25, p. 1, 2010. 10.1214/09-STS313 PMC2943670 20871802

[15] Rubin N., Thomas D. B., “Combining propensity score matching with additional adjustments for prognostic covariates,” *J*. *Am*. *Stat*. *Assoc*., vol. 450, no. 95, pp. 573–585, 2000.

[16] Normand B. J., Landrum S. L. T., Guadagnoli M. B., Ayanian E., Ryan J. Z., Cleary T. J., P. D., et al “Validating recommendations for coronary angiography following acute myocardial infarction in the elderly: a matched analysis using propensity scores,” *J*. *Clin*. *Epidemiol*., vol. 4, no. 54, pp. 387–398, 2001. 10.1016/s0895-4356(00)00321-8 11297888

[17] Rosenbaum P. R., “Heterogeneity and causality: Unit heterogeneity and design sensitivity in observational studies,” *Am*. *Stat*., vol. 2, no. 59, pp. 147–152, 2005.

[18] Mugavero M. J., Catherine R, Lesko Stephen R, Cole H, Hall Irene, Westreich Daniel, et al “The effect of antiretroviral therapy on all-cause mortality, generalized to persons diagnosed with HIV in the USA, 2009–11,” *Int*. *J*. *Epidemiol*., vol. 45, no. 1, pp. 140–150, 2016 10.1093/ije/dyv352 26772869 PMC5013889

[19] Boulle A S. B., Schomaker M, May MT, Hogg RS, “Mortality in Patients with HIV-1 Infection Starting Antiretroviral Therapy in South Africa, Europe, or North America: A Collaborative Analysis of Prospective Studies.,” *PLoS Med*, vol. 11, no. 9. 10.1371/journal.pmed.1001718 PMC4159124 25203931

[20] Brechtl J. R., Breitbart W., Galietta M., Krivo S., and Rosenfeld B., “The use of highly active antiretroviral therapy (HAART) in patients with advanced HIV infection: impact on medical, palliative care, and quality of life outcomes.,” *J*. *Pain Symptom Manage*., vol. 21, no. 1, pp. 41–51, 2001 11223313 10.1016/s0885-3924(00)00245-1

[21] Davies M.-A., Gibb D., and Turkova A., “Survival of HIV-1 vertically infected children,” *Curr*. *Opin*. *HIV AIDS*, vol. 11, no. 5, pp. 455–464, 2016 10.1097/COH.0000000000000303 27716730 PMC5384722

[22] Faizi Z., Rizwan B., Scott J., and Ahsan J., “Effectiveness of HIV Anti-retroviral Agents on the HIV/AIDS Epidemic,” *Br*. *J*. *Med*. *Med*. *Res*., vol. 15, no. 11, pp. 1–13, 2016.

[23] Reniers G., Slaymaker E., Nakiyingi-Miiro J., Nyamukapa C., Crampin A. C., Herbst K., et al “Mortality trends in the era of antiretroviral therapy,” *Aids*, vol. 28, pp. S533–S542, 2014 10.1097/QAD.0000000000000496 25406756 PMC4251911

[24] Barry O., Powell J., Renner L., Bonney E. Y., Prin M., Ampofo W., et al, “Effectiveness of first-line antiretroviral therapy and correlates of longitudinal changes in CD4 and viral load among HIV-infected children in Ghana,” 2013 10.1186/1471-2334-13-476 24119088 PMC3852953

[25] Cochran D. B., Rubin W. G., “Controlling bias in observational studies: A review,” *Sankhyā Indian J*. *Stat*., no. Series A, pp. 417–446, 1973.

[26] Austin P. C., “An introduction to propensity score methods for reducing the effects of confounding in observational studies,” *Multivariate Behav*. *Res*., vol. 3, no. 46, pp. 399–424, 2011. 10.1080/00273171.2011.568786 PMC3144483 21818162

[27] Heckman P., Ichimura J. J., Todd H., “Matching as an econometric evaluation estimator,” *Rev*. *Econ*. *Stud*., vol. 65, no. 2, pp. 261–294, 1998.

[28] Rubin N., Thomas D. B., “Matching using estimated propensity scores: relating theory to practice,” *Biometrics*, vol. 52, no. 1, pp. 249–264, 1996 8934595

